# Comparative Transcriptomic Assessment of Chemosensory Genes in Adult and Larval Olfactory Organs of *Cnaphalocrocis medinalis*

**DOI:** 10.3390/genes14122165

**Published:** 2023-11-30

**Authors:** Hai-Tao Du, Jia-Qi Lu, Kun Ji, Chu-Chu Wang, Zhi-Chao Yao, Fang Liu, Yao Li

**Affiliations:** 1 College of Plant Protection, Yangzhou University, Yangzhou 225009, China; dx120180086@stu.yzu.edu.cn (H.-T.D.); mz120221473@stu.yzu.edu.cn (J.-Q.L.); dx120230149@stu.yzu.edu.cn (K.J.); wangchuchu@jscxjszyxy1.wecom.work (C.-C.W.); 008412@yzu.edu.cn (Z.-C.Y.); 2 Jiangsu Co-Innovation Center for Modern Production Technology of Grain Crops, Yangzhou University, Yangzhou 225009, China; 3 Joint International Research Laboratory of Agriculture & Agri-Product Safety, Yangzhou University, Yangzhou 225009, China

**Keywords:** *Cnaphalocrocis medinalis*, chemosensory genes, larva, adult, transcriptome analysis, expression pattern

## Abstract

The rice leaf folder, *Cnaphalocrocis medinalis* (Lepidoptera: Pyralidae), is a notorious pest of rice in Asia. The larvae and adults of *C. medinalis* utilize specialized chemosensory systems to adapt to different environmental odors and physiological behaviors. However, the differences in chemosensory genes between the olfactory organs of these two different developmental stages remain unclear. Here, we conducted a transcriptome analysis of larvae heads, male antennae, and female antennae in *C. medinalis* and identified 131 putative chemosensory genes, including 32 *OBP*s (8 novel *OBP*s), 23 *CSP*s (2 novel *CSP*s), 55 *OR*s (17 novel *OR*s), 19 *IR*s (5 novel *IR*s) and 2 *SNMP*s. Comparisons between larvae and adults of *C. medinalis* by transcriptome and RT-qPCR analysis revealed that the number and expression of chemosensory genes in larval heads were less than that of adult antennae. Only 17 chemosensory genes (7 *OBP*s and 10 *CSP*s) were specifically or preferentially expressed in the larval heads, while a total of 101 chemosensory genes (21 *OBP*s, 9 *CSP*s, 51 *OR*s, 18 *IR*s, and 2 *SNMP*s) were specifically or preferentially expressed in adult antennae. Our study found differences in chemosensory gene expression between larvae and adults, suggesting their specialized functions at different developmental stages of *C. medinalis*. These results provide a theoretical basis for screening chemosensory genes as potential molecular targets and developing novel management strategies to control *C. medinalis*.

## 1. Introduction

The chemosensory system serves insects to cope with highly intricate and perpetually fluctuating chemical environments [1]. The insect chemosensory system relies on a diversity of proteins expressed in the chemosensory sensilla that are located on sensory organs, such as antennae and mouthparts [2]. These chemosensory proteins group several multi-gene families involved in odor reception, including two classes of ligand-binding proteins: odorant-binding proteins (OBPs) and chemosensory proteins (CSPs), and three classes of membrane receptors: odorant receptors (ORs), ionotropic receptors (IRs), and sensory neuron membrane proteins (SNMPs) [3,4]. The OBP and CSP families, two types of soluble proteins secreted in the sensory lymph, possess six or four conserved cysteines, respectively [5,6]. Two families of chemosensory receptors, namely ORs and IRs, are divergent transmembrane proteins (ORs with a seven-transmembrane domain and IRs with a four-transmembrane domain) that function as heteromeric ligand-gated ion channels with conserved co-receptors [7,8]. Additionally, SNMPs are a smaller receptor family consisting of two members, SNMP1 and SNMP2, which are characterized by two transmembrane domains [9]. These receptors have specific interactions with odorant chemicals [10]. After entering the chemosensory sensilla, external odorant chemicals are captured by ligand-binding proteins, OBPs or CSPs, and delivered to the chemosensory receptors in the dendritic membrane of olfactory sensory neurons (OSNs) [4]. The chemical signals received by the OSNs are transformed into electrical impulses, which are subsequently conveyed to the insect brain, thereby triggering behavioral responses [11].

The larvae and adults of Holometabola, with different morphologies and lifestyles, possess differentiated olfactory organs and specialized chemosensory systems for detecting environmental odors [12]. The larvae primarily forage, feed, and grow for metamorphosis, whereas the adults feed on different substrates to facilitate mate-seeking and reproduction during this developmental stage [13]. Accordingly, larvae and adults exhibit differential sensitivity to chemosensory cues and develop distinct chemosensory systems [14,15]. The larvae of *Drosophila suzukii* and *Aedes aegypti* expressed 34 *OR*s and 24 *OR*s, respectively, which is significantly lower than the number of adult *OR*s: 55 *OR*s and 84 *OR*s [16,17]. These genetic differences in chemosensory genes between larvae and adults have been verified in a few Lepidoptera species, including *Sesamia nonagrioides*, *Cydia pomonella*, *Loxostege sticticalis*, *Spodoptera littoralis*, and *Bombyx mori* [18,19,20,21,22]. Generally, the number and expression level of chemosensory genes in the olfactory organs of larvae are lower than those in adults [18,19,20,21,22]. For instance, *C. pomonella* expressed 16 *OR*s in larval heads, while a significantly higher 57 *OR*s were expressed in the adult antennae [19]. So far, only a limited number of studies have examined the differences in one or several types of chemosensory genes between larvae and adults. However, there has been a lack of systematic investigation into the divergences of the chemosensory systems between larvae and adults. 

The rice leaf folder, *C. medinalis* (Guenée), is a destructive migratory pest that causes significant damage to rice cultivation across Asia, Oceania, and Africa [23]. The larvae inflict damage on rice by folding the leaves and scraping the green leaf tissues within the fold, resulting in yield loss by reducing photosynthetic activity [24]. The transcriptome sequencing of antennae from *C. medinalis* adults has successfully identified 102 chemosensory genes, including 30 *OBP*s, 26 *CSP*s, 29 *OR*s, 15 *IR*s, and 2 *SNMP*s [25]. Another transcriptome analysis, reported in 2017, discovered more chemosensory genes, among which 37 newly discovered chemosensory genes (3 *OBP*s, 29 *OR*s, and 5 *IR*s) were identified [26]. There is a lack of systematic studies on the differences in chemosensory genes between *C. medinalis* larval and adult olfactory organs. In this study, we performed a transcriptome analysis of larval heads and male/female adult antennae from *C. medinalis*. Through the transcriptomic data analysis, we identified chemosensory genes in larval heads and adult antennae, investigated phylogenetic relationships and motifs, and compared the expression patterns of chemosensory genes between larval and adult olfactory organs.

## 2. Materials and Methods

### 2.1. Insect Specimen Preparation

The *C. medinalis* originated from a research area at Yangzhou University, Yangzhou, China. Insects were reared in a controlled laboratory environment (27 ± 1 °C, 75 ± 5% RH, and 16:8 h L:D) using rice seedlings. For analysis, samples were collected by dissecting male and female antennae from 1 to 5 days (each day-of-age moth weighing approximately 20 mg) and larvae heads of 1st to 5th instar stages (each instar weighing approximately 20 mg). For every sample, biological triplicate duplicates were carried out. The prepared samples were stored at −80 °C. 

### 2.2. Illumina Sequencing, Sequence Assembly, Function Annotation, and Expression Abundance Analysis

RNA isolation, cDNA library creation, and Illumina sequencing of the specimens were conducted at Novogene Co., Ltd. (Beijing, China). The results of Illumina sequencing filter the raw data to obtain clean reads and compare it with our *C. medinalis* genome (unpublished). The new transcript was assembled by StringTie and annotated by the Kyoto Encyclopedia of Genes and Genomes (KEGG), Gene Ontology (GO), Pfam, SUPERFAMILY, and other databases [27,28,29,30,31]. Gene expression levels were estimated using FPKM (fragments per kb of transcript sequence per million bp sequenced) values [32]. FPKM values equal to or greater than one were considered thresholds to determine if a gene was expressed. We defined FPKM > 100 as high expression, 100 > FPKM > 10 as moderate expression, and FPKM < 10 as low expression. A fold-change threshold of three was set, to identify genes with significant differential expression. Expression of chemosensory genes was revealed by heat mapping using Heml, and the expression profiles of genes were calculated based on log_e_FPKM.

### 2.3. Identification and Phylogenetic Analyses of Chemosensory Genes

The identification of unigenes associated with chemosensory genes was carried out through keyword searches of blastx annotations. Open reading frames (ORF) finder (https://www.ncbi.nlm.nih.gov/orffinder/, accessed on 7 October 2023) and blast were employed to validate the predicted protein sequences further. Signal peptides of OBPs and CSPs were predicted by SignalP-5.0 (https://services.healthtech.dtu.dk/service.php?SignalP-5.0, accessed on 19 October 2023), then the MEME-5.5.4 online server (https://meme-suite.org/meme/tools/meme, accessed on 19 October 2023) was used to discover the motifs of OBP and CSP proteins. TMHMM-2.0 (https://services.healthtech.dtu.dk/services/TMHMM-2.0/, accessed on 20 October 2023) was utilized to predict putative transmembrane domains (TMDs) in ORs, IRs, and SNMPs. The alignment of amino acid sequences encoding OBPs, CSPs, ORs, and IRs was performed using Clustal W (Supplementary Materials SA–SE). For constructing phylogenetic trees, the neighbor joining method with Poisson correction of distances was implemented in MEGA X (https://www.megasoftware.net/, accessed on 22 October 2023). Additionally, node support was evaluated through a bootstrap procedure, consisting of 1000 replicates.

### 2.4. Quantitative Real-Time Polymerase Chain Reaction Validation

To evaluate the expression patterns of chemosensory genes across various organs (same as those used in transcriptome sequencing), we employed the technique of real-time quantitative PCR (RT-qPCR). Total RNA was extracted by the Trizol method and then reverse transcribed to first-strand cDNA, using the methods provided with the One Step SYBR PrimeScript RT-PCR kit (Takara, Dalian, China). The SYBR Premix Ex Taq (Takara, Dalian, China) and the CFX96™ Real-Time PCR Detection System (Bio-Rad, Hercules, CA, USA) were used for RT-qPCR. *β-actin* was selected as the reference gene and the primers utilized for RT-qPCR were created using an online software program (https://sg.idtdna.com/Scitools/Applications/RealTimePCR/, accessed on 24 October 2023) (Table S1). The RT-qPCR was carried out using the following program: an initial denaturation step at 95 °C for 3 min, followed by 40 cycles of 10 s at 95 °C and 30 s at 60 °C [33]. The relative expression levels of specimens were calculated using the ∆∆Ct method [34]. A one-way analysis of variance was conducted in SPSS Statistics 17.0 (SPSS Inc., Chicago, IL, USA), to compare relative expression levels across various organs. A significance level of *p* < 0.05 means a statistically significant difference.

## 3. Results

### 3.1. Identification and Comparison of Odorant-Binding Proteins in C. medinalis Larval and Adult Olfactory Organs

**Figure 1:**
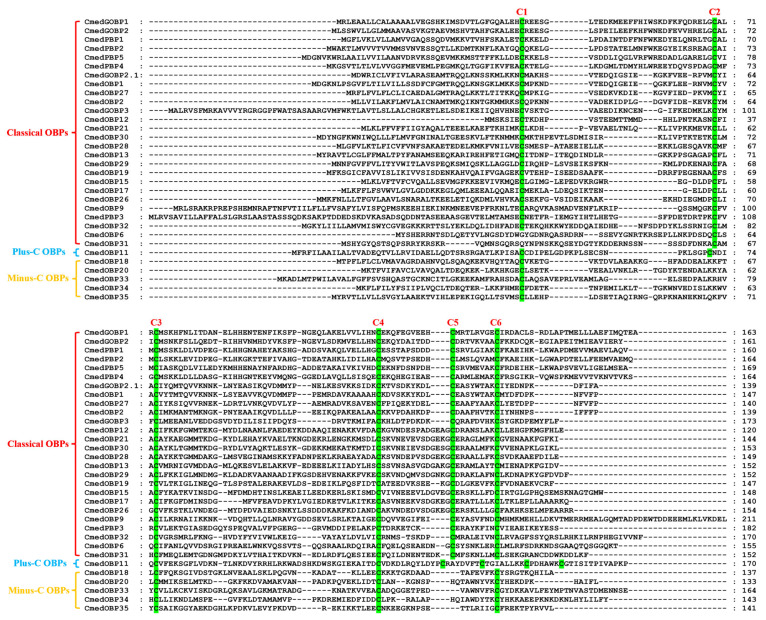
Sequences alignment of candidate CmedOBPs. The conserved cysteine residues were marked with a green shade. All these OBPs were assigned to Classical OBPs, Plus-C OBPs, and Minus-C OBPs.

**Figure 2:**
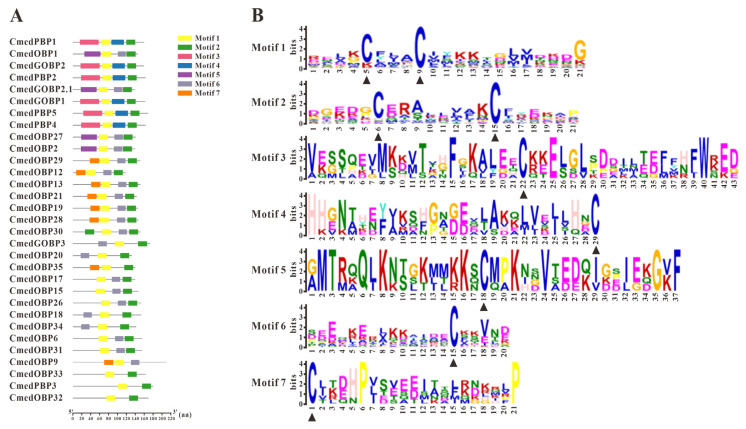
Motif analysis of candidate CmedOBPs. (**A**) Distributions of motifs in candidate CmedOBPs. The x-axis indicates the length of OBP proteins. (**B**) The SeqLogo of the motifs present in candidate CmedOBPs. The black triangle marked the conserved cysteine residues.

**Figure 3:**
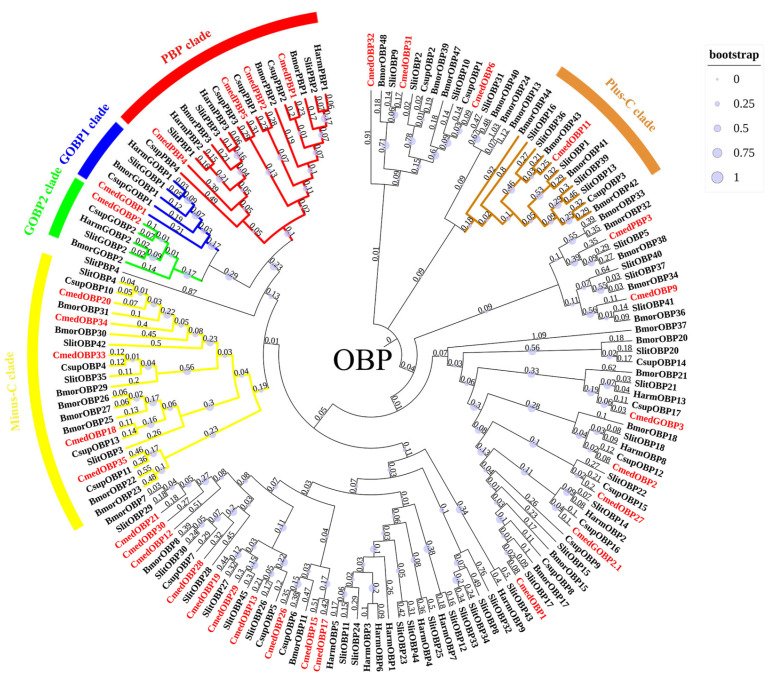
Phylogenetic tree of candidate CmedOBPs with known lepidopteran OBPs. The clade in red indicates the PBP clade; the clade in blue indicates the GOBP1 clade; the clade in green indicates the GOBP2 clade; the clade in yellow indicates the Minus-C OBPs clade; and the clade in brown indicates the Plus-C OBPs clade. The value next to the branch represents the branch length, and the size of the circle on the branch means the bootstrap value. Cmed: *C. medinalis*, Bmor: *B. mori*, Harm: *Helicoverpa armigera*, Csup: *Chilo suppressalis*, Slit: *S. littoralis*.

A total of 32 different single genes encoding hypothetical OBPs in *C. medinalis* were obtained (8 newly identified and 24 known OBPs), of which 31 *OBP* sequences contained complete ORFs and 28 OBPs possessed signal peptides (Table S2). Sequence alignment showed that most of the hypothetical OBPs shared the classical six-cysteine motifs, except for five OBPs (CmedOBP18/20/33/34/35) belonging to the Minus-C group and one (CmedOBP11) to the Plus-C group (Figure 1). To further research the characteristic regions of the OBP proteins in *C. medinalis*, we uploaded 32 OBPs to MEME and conducted a motif search. Search results showed seven motifs in the OBPs of *C. medinalis*, and all CmedOBPs had two–four motifs containing four–eight conserved cysteine residues (Figure 2). As expected, the phylogenetic tree showed that four PBPs (CmedPBP1/2/4/5) and two GOBPs (CmedGOBP1/2) clustered into PBP and GOBP clades, respectively. However, CmedPBP3 and CmedGOBP2.1/3 did not cluster into the clades corresponding to their names. In addition, all Minus-C OBPs (CmedOBP18/20/33/34/35) and Plus-C OBPs (CmedOBP11) clustered in the “Plus-C” and “Minus-C” OBP sub-families, in correlation with their cysteine number (Figure 3).

**Figure 4:**
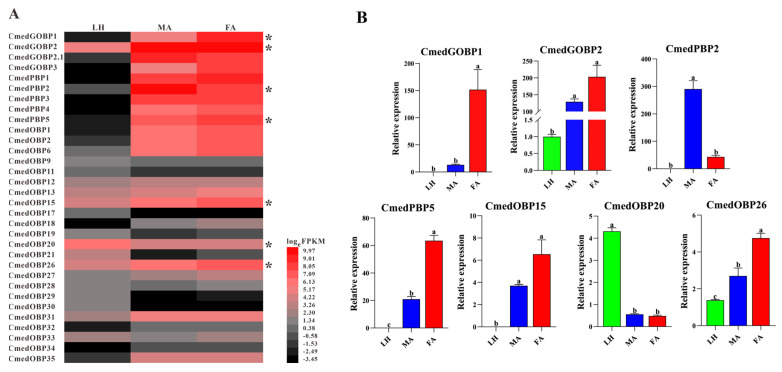
Comparison of candidate *CmedOBP*s expression in different olfactory organs of *C. medinalis*. (**A**) Heatmap based on FPKM values. Red indicates relatively higher expression while black indicates relatively lower expression. Asterisk “*” indicates expression has been validated by RT-qPCR assay. (**B**) Relative expression pattern based on RT-qPCR. The relative expression of each gene normalized to *β-actin* and *CmedGOBP2* transcript levels in larval heads were used as a baseline reference. The significant difference in different olfactory organs was marked on the bars with lowercase letters, *p* < 0.05. LH: larval heads, MA: male antennae, FA: female antennae.

**Figure 5:**
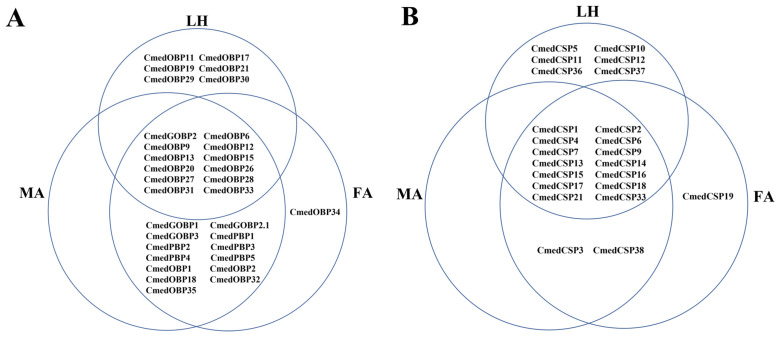
Distribution of *CmedOBP*s and *CmedCSP*s in different olfactory organs of *C. medinalis.* (**A**) *CmedOBP*s. (**B**) *CmedCSP*s. LH: larval heads, MA: male antennae, FA: female antennae.

To reveal the difference of *CmedOBP*s expression between larvae and adults, the expression profiles of 32 *OBP*s in larval heads and adult antennae were compared via heatmap analysis. RNA-Seq results revealed the quantity and transcription level of *CmedOBP*s in different olfactory organs. There were 18, 25, and 26 *OBP*s identified in larval heads and male and female antennae, respectively. Six *OBP*s (*CmedOBP*11/17/19/21/29/30) showed larval head-specific expression. At the same time, 14 *OBP*s expressed exclusively in adult antennae, of which *CmedOBP34* was detected only in female antennae (Figure 4A and Figure 5A). Among the *OBP*s expressed in both the larval heads and adult antennae, *CmedOBP20* displayed larval head-biased expression, while seven *OBP*s (*CmedGOBP2*, *CmedOBP6*/*13*/*15*/*26*/*27*/*31*) exhibited preferential expression in adult antennae. In addition, three *OBP*s (*CmedGOBP1*, *CmedPBP2*/*5*) showed gender differential expression (Figure 4A). 

We investigated the expression patterns of four *OBP*s (*CmedGOBP2*, *CmedOBP15*/*20*/*26*) that exhibited high mRNA levels in larval heads and three *OBP*s (*CmedGOBP1*, *CmedPBP2*/*5*) that displayed gender differential expression by RT-qPCR analysis to confirm the data of transcriptomes. RT-qPCR results showed that the expression levels of *CmedGOBP1* and *CmedPBP5* in female antennae were 1038.4% and 203.2% higher than those in male antennae, respectively. Meanwhile, the expression level of *CmedPBP2* in male antennae was 454.5% higher than in female antennae. In addition, the expression of *CmedOBP20* in larval heads was 798.2% and 678.6% higher than that in female and male antennae, respectively, indicating that *CmedOBP20* expressed preferentially in larval heads. Unexpectedly, *CmedOBP15* was not detected in larval heads by RT qPCR (Figure 4B). 

### 3.2. Identification and Comparison of Chemosensory Proteins in C. medinalis Larval and Adult Olfactory Organs

**Figure 6:**
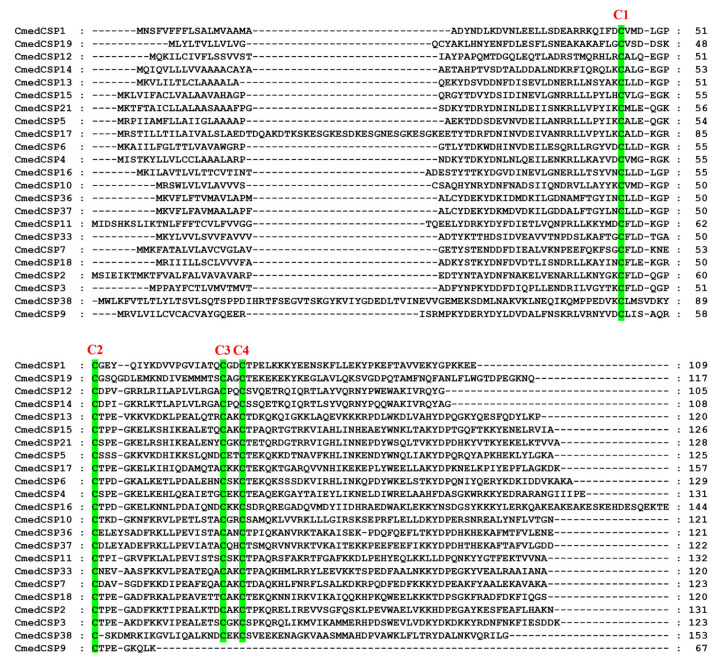
Sequences alignment of candidate CmedCSPs. The conserved cysteine residues were marked with a green shade.

**Figure 7:**
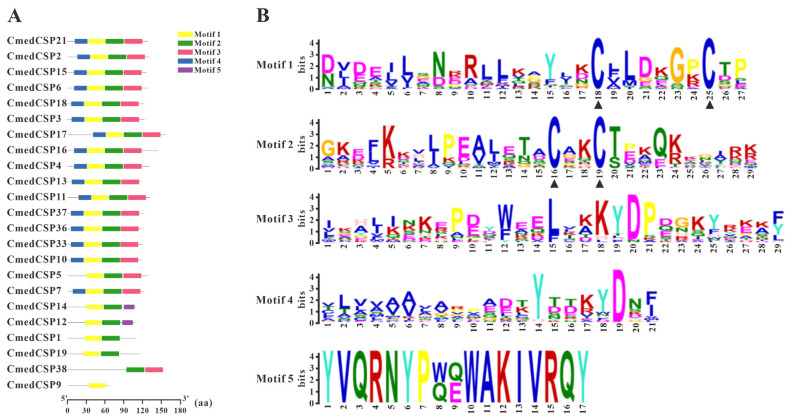
Motif analysis of candidate CmedCSPs. (**A**) Distributions of motifs in candidate CmedCSPs. The x-axis indicates the length of CSP proteins. (**B**) The SeqLogo of the motifs present in candidate CmedCSPs. The black triangle marked the conserved cysteine residues.

**Figure 8:**
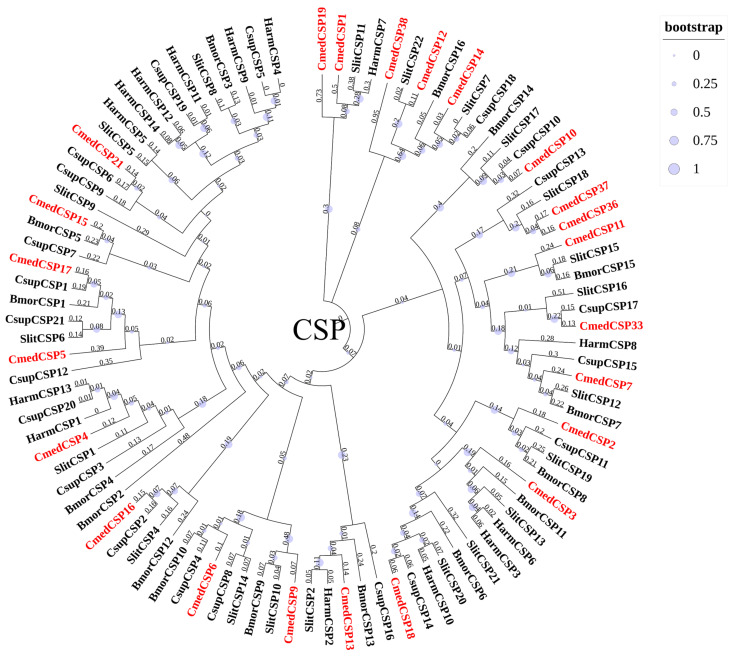
Phylogenetic tree of candidate CmedCSPs with known lepidopteran CSPs. The value next to the branch represents the branch length, and the size of the circle on the branch means the bootstrap value. Cmed: *C. medinalis*, Bmor: *B. mori*, Harm: *H. armigera*, Csup: *C. suppressalis*, Slit: *S. littoralis*.

According to transcripts annotation, we identified 23 *CSP*s from *C. medinalis* larval and adult transcriptomes, including 2 novel *CSP*s (*CmedCSP37*/*38*) and 21 known *CSP*s. Except for *CmedCSP9*, 22 *CSP*s had full-length ORFs (Table S3). The pattern of four conserved cysteine residues was retained among the 22 candidate CSPs, except the remaining CmedCSP9 sequence was too short (Figure 6). Motif analysis using MEME revealed five motifs in 23 putative CSPs, of which two highly conserved motifs (Motif1/2) contained four conserved cysteine residues (Figure 7). No subfamilies were distinguished in the phylogenetic tree, but most CmedCSPs clustered with other Lepidoptera CSPs (Figure 8).

**Figure 9:**
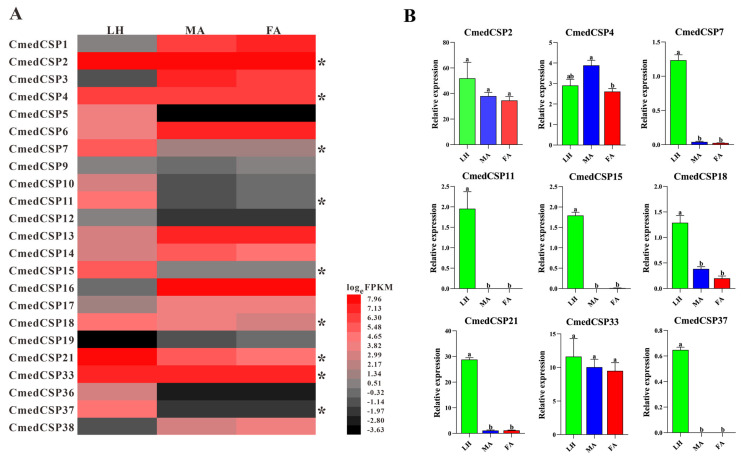
Comparison of candidate *CmedCSP*s expression in different olfactory organs of *C. medinalis*. (**A**) Heatmap based on FPKM values. Red indicates relatively higher expression while black indicates relatively lower expression. Asterisk “*” indicates expression has been validated by RT-qPCR assay. (**B**) Relative expression pattern based on RT-qPCR. The relative expression of each gene normalized to *β-actin* and *CmedGOBP2* transcript levels in larval heads were used as a baseline reference. The significant difference in different olfactory organs was marked on the bars with lowercase letters, *p* < 0.05. LH: larval heads, MA: male antennae, FA: female antennae.

The number of *CSP*s expressed in larval heads and male and female antennae were 20, 16, and 17, respectively. Transcriptome results showed that six *CSP*s (*CmedCSP5*/*10*/*11*/*12*/*36*/*37*) specifically expressed in larval heads, while three *CSP*s (*CmedCSP3*/*19*/*38*) showed exclusive expression in adult antennae. In addition, *CmedCSP19* exhibited specifical expression in female antennae (Figure 5B and Figure 9A). The transcriptome results also revealed that four *CSP*s (*CmedCSP7*/*15*/*18*/*21*) displayed larval head-biased expression. In comparison, six *CSPs* (*CmedCSP1*/*6*/*13*/*14*/*16*/*17*) were expressed at higher levels in adult antennae. No sex-differentially expressed *CSP*s were found (Figure 9A). The transcript expression level of nine highly expressed *CSP*s (*CmedCSP2*/*4*/*7*/*11*/*15*/*18*/*21*/*33*/*37*) in larval heads were validated by RT-qPCR. Qualitative data revealed that four *CSP*s (*CmedCSP7*/*11*/*15*/*37*) were detected only in larval heads, and there was no significant difference in the expression levels of three *CSP*s (*CmedCSP2*/*4*/*33*) in larval heads compared to adult antennae. Moreover, the expression levels of two *CSP*s (*CmedCSP18*/*21*) in larval heads were 235.4%/2469.7% higher than male antennae and 544.8%/2304.4% higher than female antennae, respectively (Figure 9B).

### 3.3. Identification and Comparison of Odorant Receptors in C. medinalis Larval and Adult Olfactory Organs

**Figure 10:**
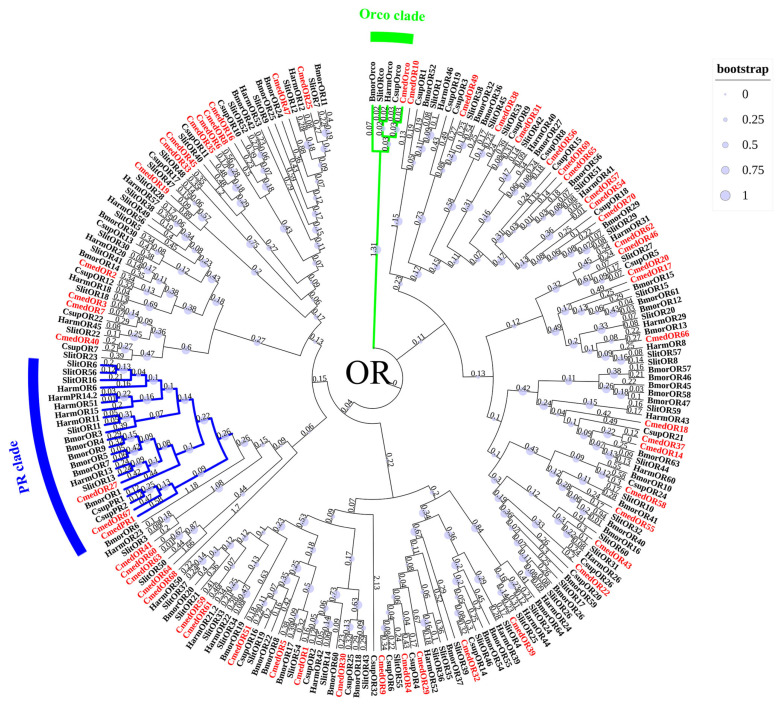
Phylogenetic tree of candidate CmedORs with known lepidopteran ORs. The clade in green indicates the Orco clade; the clade in blue indicates the PR clade. The value next to the branch represents the branch length, and the size of the circle on the branch means the bootstrap value. Cmed: *C. medinalis*, Bmor: *B. mori*, Harm: *H. armigera*, Csup: *C. suppressalis*, Slit: *S. littoralis*.

Transcriptome analysis of *C. medinalis* olfactory organs revealed 55 candidate *OR*s, of which 17, newly identified, were named *CmedOR54*–*CmedOR70* (Table S4). The complete ORFs of 48 *OR*s were predicted, and the ORFs of 7 *OR*s (*CmedOR32*/*45*/*47*/*57*/*61*/*62*/*69*) were missing either the 5′ terminal or 3′ terminal. Eleven ORs were expected to contain the seven TMDs, while the other ORs had zero–six TMDs. The phylogenetic tree revealed the relationship between CmedORs and other ORs from Lepidoptera species (*B. mori*/*H. armigera*/*C. suppressalis*/*S. littoralis*) (Figure 10). A highly conserved Orco clade contained CmedOrco and Orcos from other Lepidoptera species. In addition, CmedPR1, CmedOR27, and CmedOR67 clustered with the moths’ PR subfamily.

**Figure 11:**
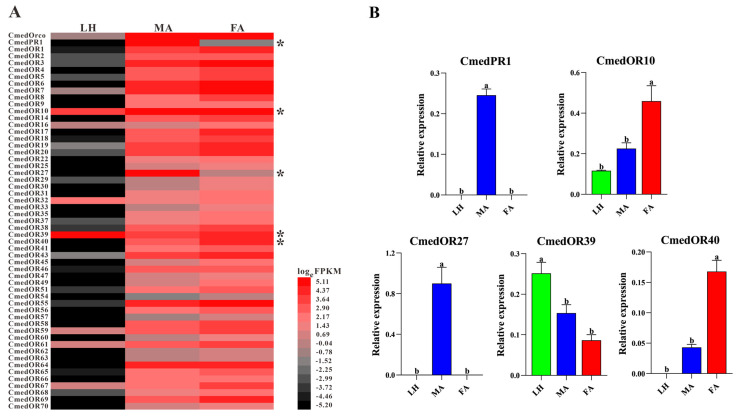
Comparison of candidate *CmedOR*s expression in different olfactory organs of *C. medinalis*. (**A**) Heatmap based on FPKM values. Red indicates relatively higher expression while black indicates relatively lower expression. Asterisk “*” indicates expression has been validated by RT-qPCR assay. (**B**) Relative expression pattern based on RT-qPCR. The relative expression of each gene normalized to *β-actin* and *CmedGOBP2* transcript levels in larval heads were used as a baseline reference. The significant difference in different olfactory organs was marked on the bars with lowercase letters, *p* < 0.05. LH: larval heads, MA: male antennae, FA: female antennae.

**Figure 12:**
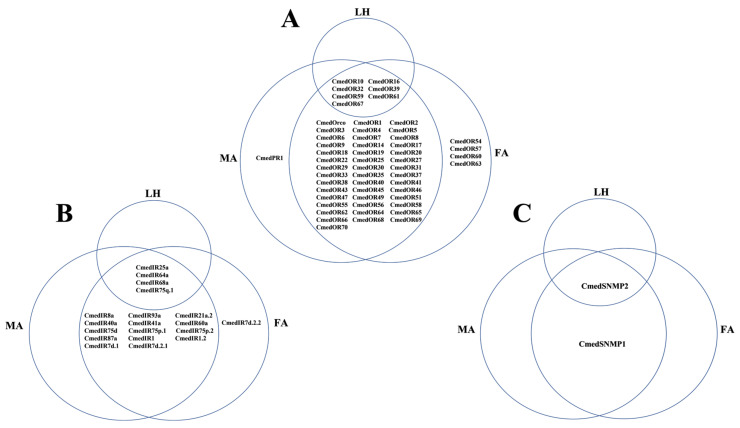
Distribution of *CmedSNMP*s, *CmedOR*s, and *CmedIR*s in different olfactory organs of *C. medinalis*. (**A**) *CmedOR*s, (**B**) *CmedIR*s, (**C**) *CmedSNMP*s. LH: larval heads, MA: male antennae, FA: female antennae.

The transcriptome-guided read-mapping showed that 7, 51, and 54 *OR*s were expressed in larval heads and male and female antennae, respectively. There were no specific expression *OR*s in larval heads, while 48 *OR*s exhibited exclusive expression in adult antennae. *CmedPR1* exhibited specifical expression in male antennae, and four *OR*s (*CmedOR54*/*57*/*60*/*63*) showed female antennae-specifical expression (Figure 11A and Figure 12A). In addition, three *OR*s (*CmedOR59*/*61*/*67*) had lower expression levels in larval heads than adult antennae (Figure 11A). Two moderately expressed *OR*s (*CmedOR10*/*39*) and three gender differential expression *OR*s (*CmedPR1*/*CmedOR27*/*40*) were validated through RT-qPCR analysis. The results revealed that *CmedPR1* and *CmedOR27* were specifically expressed in male antennae, and the expression level of *CmedOR40* in female antennae was 290.0% higher than that in male antennae. According to RT-qPCR results, the expression level of *CmedOR39* in larval heads was significantly higher than that in adult antennae (Figure 11B).

### 3.4. Identification and Comparison of Ionotropic Receptors and Sensory Neuron Membrane Proteins in C. medinalis Larval and Adult Olfactory Organs

**Figure 13:**
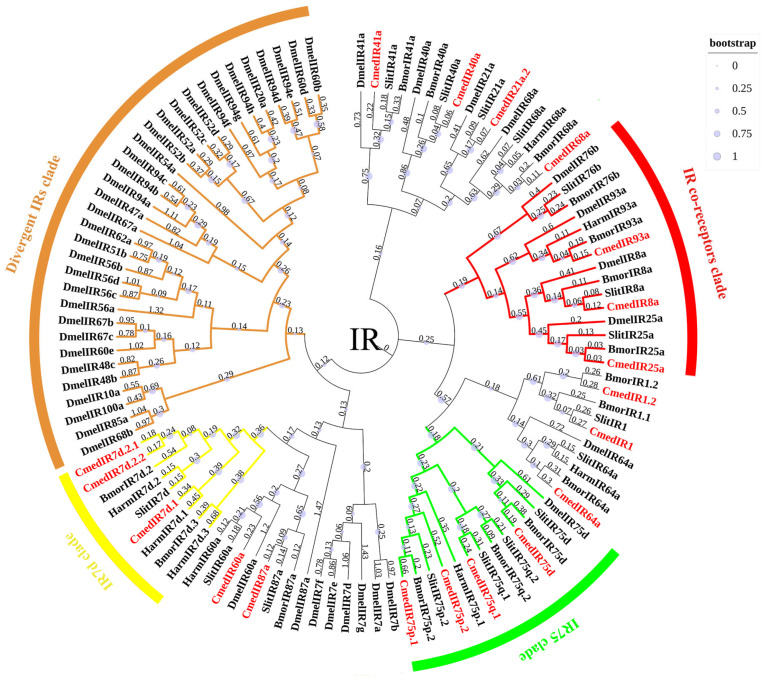
Phylogenetic tree of candidate CmedIRs with known insect’s IRs. The clade in red indicates the IR co-receptors clade; the clade in green indicates the IR75 clade; the clade in yellow indicates the IR7d clade; the clade in brown indicates the divergent IRs clade. The value next to the branch represents the branch length, and the size of the circle on the branch means the bootstrap value. Cmed: *C. medinalis*, Bmor: *B. mori*, Harm: *H. armigera*, Slit: *S. littoralis*, Dmel: *D. melanogaster*.

**Figure 14:**
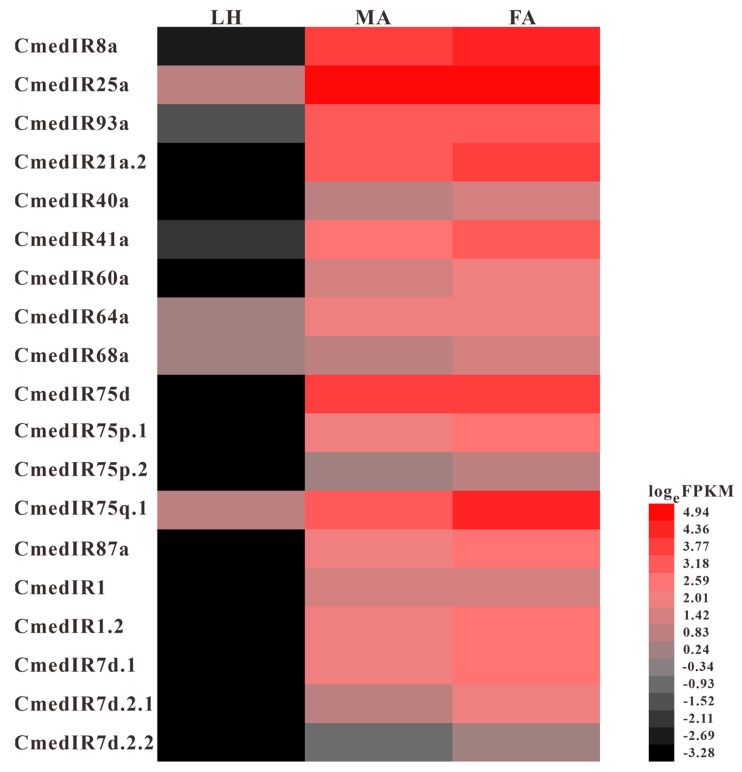
Comparison of candidate *CmedIR*s expression based on FPKM values in different olfactory organs of *C. medinalis*. Red indicates relatively higher expression while black indicates relatively lower expression. LH: larval heads, MA: male antennae, FA: female antennae.

Nineteen *IR*s were identified in *C. medinalis* and were named based on their blastx best hits to insect IRs and their positions in the phylogenetic tree, including 5 new *IR*s and 14 known *IR*s (Table S5). Of these, 18 *IR*s contained full-length ORFs, except *CmedIR40a*, which lacked a complete 5′ terminal. All hypothetical IRs in *C. medinalis* were confirmed to have one–four TMDs. A phylogenetic tree, indicating evolutionary relationships between *C. medinalis* IRs and those from *Drosophila melanogaster* and other Lepidoptera (*B. mori*/*H. armigera*/*C. suppressalis*/*S. litura*), is shown (Figure 13). This tree indicates that most CmedIRs were separated from each other and clustered in a branch with their homologous genes. A highly conserved IR co-receptors clade and the two large sub-families of IR7d and IR75 clades were present here. *D. melanogaster* divergent IRs clustered phylogenetically into separate clades. According to their phylogenetic position, five new sequences were named “CmedIR1”, “CmedIR1.2”, “CmedIR7d.1”, “CmedIR7d.2.1”, and “CmedIR7d.2.2”. Transcripts encoding 4, 18, and 19 IRs were identified in larval heads and male and female antennas, respectively. The RNA-Seq results also revealed that 15 *IR*s expressed specifically in adult antennae, in which *CmedIR7d.2.2* was specifically expressed in female antennae. In addition, three *IR*s (*CmedIR25a*/*64a*/*75q.1*) showed lower expression levels in larval heads than adult antennae (Figure 12B and Figure 14).

Two previously reported *CmedSNMP*s, *CmedSNMP*1 and *CmedSNMP*2, were identified in our *C. medinalis* transcriptome (Table S6). These two *SNMP*s have complete ORFs and display 100 and 98.85 percent identity to the previously reported sequences at the protein level. Only *CmedSNMP*2 with low mRNA levels (FPKM = 4.58) was detected in larval heads, and the expression level was significantly lower than that in male antennae (FPKM = 1401.96) and female antennae (FPKM = 1314.23) (Figure 12C).

## 4. Discussion

Using transcriptomic analysis, we identified 131 chemosensory genes from the olfactory organs of *C. medinalis*, including 32 *OBP*s, 23 *CSP*s, 55 *OR*s, 19 *IR*s, and 2 *SNMP*s. The number of chemosensory genes exhibits a comparable magnitude in other lepidopteran species, such as *S. littoralis* (122 chemosensory genes), *C. suppressalis* (116 chemosensory genes), *H. armigera* (139 chemosensory genes), *Helicoverpa assulta* (131 chemosensory genes), and *L. sticticalis* (112 chemosensory genes) [20,35,36,37]. In previous studies, Zeng and Liu discovered 102 and 90 chemosensory genes in *C. medinalis* adults, respectively [25,26]. More chemosensory genes were identified in our study, including eight novel *OBP*s, two novel *CSP*s, seventeen novel *OR*s, and five novel *IR*s. This may be attributed to transcriptome sequencing analysis referring to our *C. medinalis* genome (Unpublished) and the addition of larval head samples. Further validation is required for the sequence and expression of chemosensory genes in *C. medinalis*.

The studies in most Lepidoptera species demonstrated that the number and expression level of *OBP*s in larval olfactory organs is comparatively lower than those found in adult antennae [18,38,39]. The larvae of *H. armigera* expressed a total of 26 *OBP*s in antennae and mouthparts, while the adults exhibited the expression of 34 *OBP*s in their antennae, of which 6 and 10 *OBP*s showed specific or biased expression in larvae and adults, respectively [38]. An even more striking phenomenon was observed in *S. nonagrioides*, where only *SnonOBP3*, among the identified 12 *OBP*s, showed preferential expression in the antennae and palps of larvae [18]. Two studies in *Spodoptera exigua* produced opposite results in the comparison of specifically or biasedly expressed *OBP*s in larvae and adults [39,40]. The truth in *S. exigua* needs further research. In our study, 18 *CmedOBP*s were identified in *C. medinalis* larval heads, and 26 *CmedOBP*s were identified in the antennae of adults. Seven *CmedOBP*s showed higher expression levels in larval heads compared to those in adult antennae, whereas twenty-one *CmedOBP*s exhibited opposite expression preference. Our results, similar to findings in other Lepidoptera species [18,38], demonstrated that larvae only need a small amount of OBPs to execute the corresponding behaviors and life activities during this development stage. 

Studies conducted in *B. mori*, *S. littoralis*, and *S. nonagrioides* found that larvae and adults expressed a similar number of *CSP*s in their olfactory organs [18,21,41]. However, the expression patterns of *CSP*s in adults and larvae exhibited variations among different Lepidoptera species. For instance, the larval heads of *B. mori* expressed eight *CSP*s (*BmorCSP10* showed specific expression in larvae) versus eleven *CSP*s in the adult antennae (four *CSP*s were expressed specifically in adults) [41]. Seventeen *CSP*s of *S. littoralis* presented an overlapping expression between larval and adult chemosensory organs, with only *SlitCSP21* being specifically expressed in larval antennae and palps [21]. In *S. nonagrioides*, thirteen *CSP*s were expressed in larval antennae/palps and eleven *CSP*s were detected in adult antennae, with six and five of them displaying larval-specific/biased or adult-specific/biased expression, respectively [18]. Similarly, larval heads and adult antennae of *C. medinalis* have a comparable magnitude of *CSP*s (larvae expressed 20 *CSP*s versus 17 in adults). Moreover, ten *CSP*s were specifically or preferentially expressed in larval heads, and nine *CSP*s have higher expression levels in adult antennae. Our studies and other reports on various insects indicate that *CSP*s are expressed throughout the insect body and across development stages, implying that they may participate in other physiological processes beyond chemosensory processes [42]. The differences in *CSP* expression patterns suggest their specialized functions at different developmental stages.

The larval *OR* repertoires are much smaller than adult ones. In Lepidoptera, *C. pomonella* larval heads expressed 16 *OR*s versus 57 *OR*s in adult antennae [19], and *S. exigua* larvae expressed 1 ORs versus 7 in adults [39]. In Diptera, the 4 *OR*s detected in *D. suzukii* larval heads were far lower than the 50 *OR*s present in adult antennae (FPKM values greater than one were considered thresholds to determine if a gene was expressed) [16]. Our results showed that the number of *OR*s detected in larval heads of *C. medinalis* was limited to only 7, which is significantly fewer than 55 *OR*s expressed in adult antennae. Like *S. littoralis*, *S. exigua*, and *S. nonagrioides*, no specific expressed *OR*s were detected in larval heads of *C. medinalis*, based on transcriptome results [18,21,40]. All these studies reveal that Lepidoptera and Diptera larvae possess a simpler olfactory system, with a lower number of *OR*s expressed, and have some overlap with adult *OR*s. This may be related to the fact that adults need to undertake more life activities than larvae, which requires more experimental support.

The abundance of other membrane receptors, *IR*s and *SNMP*s, in insect larvae is also significantly lower than in adults. For instance, nine of the ten *IR*s of *S. nonagrioides* were female antennal specifically/biasedly expressed [18]. Compared with 1 *IR* identified from the *D. suzukii* larvae heads, 28 *IR*s were identified from adult antennae (FPKM values greater than one were considered thresholds to determine if a gene was expressed) [16]. Our transcriptome analysis also found that eighteen of the nineteen *CmedIR*s exhibited specific or preferential expression in *C. medinalis* adult antennae, while only four *IR*s showed low expression levels in larval heads. Interestingly, an IR co-receptor, *CmedIR25a*, was detected in low expression in *C. medinalis* larval heads. This mirrors the observed pattern of *IR*s in *S. exigua* and *S. nonagrioides*, suggesting that IR co-receptors play a vital role in the larval heads [18,39]. In addition, *SNMP*1 and *SNMP*2 were specifically and preferentially expressed in *C. medinalis* adult antennae, respectively, like *SNMP*s in *S. exigua* and *S. nonagrioides* [18,39]. Considering that there are few studies on the functions of IRs and SNMPs in Lepidoptera larvae, we speculate that IRs and SNMPs may perform more functions in the chemosensory processes of adults than those of larvae. Further exploration of IR and SNMP functions is warranted.

## 5. Conclusions

This study presented a comprehensive investigation of 131 chemosensory genes in the olfactory organs of *C. medinalis*. The comparison of the expression profiles of 131 chemosensory genes revealed differences between larval and adult chemosensory systems, which can be attributed to their different physiological and biochemical characteristics during the development stages. Our findings identified a series of larva or adult-specific chemosensory genes that may be important for developmental stage-specific chemosensory behaviors. These chemosensory genes could serve as potential molecular targets for *C. medinalis* control in different developmental stages.

## Data Availability

Transcriptome data in this study have been uploaded to NCBI Sequence Read Archive (SRA) under accession number PRJNA1039023; all sequences (accession on Supplementary Materials SA–SE) have been uploaded.

## Supplementary Materials

The following supporting information can be downloaded at: https://www.mdpi.com/article/10.3390/genes14122165/s1, Table S1: oligonucleotide primers used for expression validation analysis; Table S2: list of candidate OBPs in *C. medinalis*; Table S3: list of candidate CSPs in *C. medinalis*; Table S4: list of candidate ORs in *C. medinalis*; Table S5: list of candidate IRs in *C. medinalis*; Table S6: list of candidate SNMPs in *C. medinalis*. Supplementary Materials SA: amino acid sequences of OBPs, CSPs, ORs, IRs, and SNMPs in *C. medinalis.* Supplementary Materials SB: amino acid sequences of OBPs used in phylogenetic analyses. Supplementary Materials SC: amino acid sequences of CSPs used in phylogenetic analyses. Supplementary Materials SD: amino acid sequences of ORs used in phylogenetic analyses. Supplementary Materials SE: amino acid sequences of IRs used in phylogenetic analyses.
