# Supplementary material for: Comparative Transcriptomic Assessment of Chemosensory Genes in Adult and Larval Olfactory Organs of Cnaphalocrocis medinalis
Source: Genes (Basel). 2023 Nov 30;14(12):2165. doi: 10.3390/genes14122165 (PMC10742765; doi:10.3390/genes14122165)
Supplement: Supplementary file 1 [file genes-14-02165-s001.zip › Supplementary Materialsú¿Tables S1-S6ú¬.pdf]

Supplementary Materials

Table S1. Oligonucleotide primers used for expression validation analysis.

Gene name	Forward primer sequence	Reverse primer sequence
β-Actin	CGAGCGTGGTTACTCATTCA	ATGACTTCTCGAGCGAGCTG
CmedGOBP1	CGACAGAGTTCATAGTGGCTC	ACATCAAGCAGTGGTCTTCG
CmedGOBP2	GCCATGTAACAGCTCACTTTG	AGTCCTCGTTCCAGAAATGC
CmedPBP2	TGGAGCAGATGACGAAACG	TTGAGCTTGTGTATCTCGTCTC
CmedPBP5	TGGAGCAGATGACGAAACG	TTGAGCTTGTGTATCTCGTCTC
CmedOBP15	GGAATGTTTGGGCATAATGGG	AAACACGCAAGGAAACAAGG
CmedOBP20	GCGTTGGTAAACAAGCTCAG	AGCACTTCACGTAGTTCCAAG
CmedOBP26	TGCGTCTTCAAGAGTACCAAG	TGCTGTCTTCAATCGCTACG
CmedCSP2	ATGACAACCTGAACCTCCAAG	AGTGCTCTTTCAACTCCTTTCC
CmedCSP4	ATGACAACCTGAACCTCCAAG	AGTGCTCTTTCAACTCCTTTCC
CmedCSP7	TCGCAACAGCCTTAGTTCTC	TCATTCTTGTCCAGGAAGCAG
CmedCSP11	TGACATTAACGAGGTGCTGG	GGCGCACTGAGTTTCTAGAG
CmedCSP15	CAGCACCAAATACGACAACTTC	AGAAGCAGTTGATGTACGCC
CmedCSP18	CAGCACCAAATACGACAACTTC	AGAAGCAGTTGATGTACGCC
CmedCSP21	GCTCTGACAAGTACACCGAC	GATCTAAGTTCCTTGCCCTCAG
CmedCSP33	AGTTGCCGACACGTACAAG	GCAGCGACTTCATTACAAGC
CmedCSP37	GCCAACACTGCACATCAATG	CCAAGACGAACGCTGTAAAAG
CmedPR1	GACCACTTCTACTTCACGAGC	GTCTTCACCAGGACCTTGATG
CmedOR10	CCCTGCCCTTTGACTACATG	TAGGAGATGTAGCAGTTGAATGAG
CmedOR27	CCCTGCCCTTTGACTACATG	TAGGAGATGTAGCAGTTGAATGAG
CmedOR39	ATATCTGACGCATGTTGGGAG	GAGACCTCTTCAAATCCTGGG
CmedOR40	ATATCTGACGCATGTTGGGAG	GAGACCTCTTCAAATCCTGGG

Table S2. List of candidate odorant binding proteins in *C. medinalis.*

Gene name	Length (bp)	ORF (aa)	Accession number	Status	Signal peptide	BLASTX best hit (Acc. no./Gene name/species)	E-value	Identity(%)
CmedGOBP1	492	163	AFG72996	complete ORF	19	gb|AFG72996.1|general odorant binding protein 1 [*Cnaphalocrocis medinalis*]	2e-103	100
CmedGOBP2	486	161	AFG72997	complete ORF	20	gb|AFG72997.1|general odorant binding protein 2 [*Cnaphalocrocis medinalis*]	8e-116	100
CmedGOBP2.1	420	139	AGI37366	complete ORF	18	gb|AGI37366.1| general odorant-binding protein 2 [*Cnaphalocrocis medinalis*]	4e-83	100
CmedGOBP3	423	140	AGI37362	complete ORF	18	gb|AGI37362.1| general odorant-binding protein 3 [*Cnaphalocrocis medinalis*]	5e-98	99
CmedPBP1	483	160	AFG72999	complete ORF	18	gb|AFG72999.1| pheromone-binding protein 1 [*Cnaphalocrocis medinalis*]	3e-111	98
CmedPBP2	495	164	AGI37364	complete ORF	21	gb|AGI37364.1| pheromone binding protein 2 [*Cnaphalocrocis medinalis*]	4e-102	100
CmedPBP3	549	182	AGI37367	complete ORF	22	gb|AGI37367.1| pheromone binding protein 3 [*Cnaphalocrocis medinalis*]	2e-127	98
CmedPBP4	495	164	AGI37368	complete ORF	21	gb|AGI37368.1| pheromone binding protein 4 [*Cnaphalocrocis medinalis*]	3e-95	99
CmedPBP5	510	169	ALT31680	complete ORF	26	gb|ALT31680.1| pheromone binding protein 5 [*Cnaphalocrocis medinalis*]	4e-111	99
CmedOBP1	444	147	AFG72998	complete ORF	26	gb|AFG72998.1| odorant-binding protein 1 [*Cnaphalocrocis medinalis*]	1e-101	96
CmedOBP2	420	139	AFG73000	complete ORF	18	gb|AFG73000.1| odorant-binding protein 2 [*Cnaphalocrocis medinalis*]	9e-96	99
CmedOBP6	468	155	ALT31636	complete ORF	NO	gb|ALT31636.1| odorant-binding protein 6 [*Cnaphalocrocis medinalis*]	7e-155	99
CmedOBP9	636	211	ALT31639	complete ORF	NO	gb|ALT31639.1| odorant-binding protein 9 [*Cnaphalocrocis medinalis*]	7e-150	100
CmedOBP11	513	170	ALT31641	complete ORF	15	gb|ALT31641.1| odorant-binding protein 11 [*Cnaphalocrocis medinalis*]	3e-121	100
CmedOBP12	363	120	ALT31642	complete ORF	NO	gb|ALT31642.1| odorant-binding protein 12 [*Cnaphalocrocis medinalis*]	1e-80	99
CmedOBP13	453	150	ALT31643	complete ORF	22	gb|ALT31643.1| odorant-binding protein 13 [*Cnaphalocrocis medinalis*]	4e-109	100
CmedOBP15	447	148	ALT31645	complete ORF	20	gb|ALT31645.1| odorant-binding protein 15 [*Cnaphalocrocis medinalis*]	5e-103	100
CmedOBP17	423	140	ALT31647	complete ORF	16	gb|ALT31647.1| odorant-binding protein 17 [*Cnaphalocrocis medinalis*]	2e-68	100
CmedOBP18	414	137	ALT31648	complete ORF	18	gb|ALT31648.1| odorant-binding protein 18 [*Cnaphalocrocis medinalis*]	5e-84	100
CmedOBP19	444	147	ALT31649	3′ missing	20	gb|ALT31649.1| odorant-binding protein 19 [*Cnaphalocrocis medinalis*]	2e-76	100
CmedOBP20	402	133	ALT31650	complete ORF	16	gb|ALT31650.1| odorant-binding protein 20 [*Cnaphalocrocis medinalis*]	4e-92	99
CmedOBP21	435	144	ALT31651	complete ORF	16	gb|ALT31651.1| odorant-binding protein 21 [*Cnaphalocrocis medinalis*]	2e-75	100
CmedOBP26	465	154	APY22693	complete ORF	22	gb|APY22693.1| odorant binding protein 26 [*Cnaphalocrocis medinalis*]	7e-107	100
CmedOBP27	423	140	APY22694	complete ORF	19	gb|XP_028178754.1| general odorant-binding protein 19a-like [*Ostrinia furnacalis*]	8e-62	71
CmedOBP28*	450	149	novel.2164	complete ORF	21	gb|APG32538.1| odorant binding preotein [*Conogethes punctiferalis*]	9e-57	68
CmedOBP29*	459	152	novel.2165	complete ORF	19	gb| UVB79204.1 | odorant-binding protein 14 [*Heortia vitessoides*]	6e-59	64
CmedOBP30*	462	153	novel.3604	complete ORF	25	gb| XP_028034646.1| uncharacterized protein LOC114246361 [*Bombyx mandarina*]	1e-44	59
CmedOBP31*	459	152	evm.TU.000022F.10	complete ORF	NO	gb| ALC76544.1| odorant binding protein 4 [*Conogethes punctiferalis*]	7e-87	86

CmedOBP32*	513	170	evm.TU.000084F.49	complete ORF	22	gb|RVE52919.1|hypothetical protein evm_002396 [*Chilo suppressalis*]	9e-07	32
CmedOBP33*	495	164	evm.TU.000000F.66	complete ORF	24	gb| APG32531.1| odorant binding preotein [*Conogethes punctiferalis*]	2e-100	87
CmedOBP34*	432	143	evm.TU.000322F.8	complete ORF	17	gb|AFG72996.1|general odorant binding protein 1 [*Cnaphalocrocis medinalis*]	6e-61	62
CmedOBP35*	426	141	evm.TU.000490F.23	complete ORF	17	gb|AFG72997.1|general odorant binding protein 2 [*Cnaphalocrocis medinalis*]	1e-61	64

Note: The newly identified CmedOBPs are marked with *.

Table S3. List of candidate chemosensory proteins in *C. medinalis.*

Gene name	Length (bp)	ORF (aa)	Accession number	Status	Signal peptide	BLASTX best hit (Acc. no./Gene name/species)	E-value	Identity(%)
CmedCSP1	330	109	AGI37361	complete ORF	18	gb|AGI37361.1|chemosensory protein 1 [*Cnaphalocrocis medinalis*]	3e-72	99
CmedCSP2	396	131	AGI37363	complete ORF	23	gb| AGI37363.1| chemosensory protein 2 [*Cnaphalocrocis medinalis*]	2e-75	100
CmedCSP3	372	123	AGI37365	complete ORF	17	gb|AGI37365.1| chemosensory protein 3 [*Cnaphalocrocis medinalis*]	2e-70	100
CmedCSP4	396	131	AIX97823	complete ORF	18	gb|AIX97823.1| chemosensory protein [*Cnaphalocrocis medinalis*]	4e-80	99
CmedCSP5	378	125	AIX97824	complete ORF	18	gb|AIX97824.1| chemosensory protein [*Cnaphalocrocis medinalis*]	1e-70	99
CmedCSP6	390	129	AIX97825	complete ORF	18	gb|AIX97825.1| chemosensory protein [*Cnaphalocrocis medinalis*]	2e-82	100
CmedCSP7	372	123	AIX97826	complete ORF	19	gb|AIX97826.1| chemosensory protein [*Cnaphalocrocis medinalis*]	2e-69	99
CmedCSP9	201	66	AIX97828	3′ missing	16	gb|AIX97828.1| chemosensory protein [*Cnaphalocrocis medinalis*]	2e-30	100
CmedCSP10	366	121	AIX97829	complete ORF	17	gb|AIX97829.1| chemosensory protein [*Cnaphalocrocis medinalis*]	1e-75	100
CmedCSP11	399	132	AIX97830	complete ORF	25	gb|QIJ45722.1| chemosensory protein [*Glyphodes pyloalis*]	7e-63	72
CmedCSP12	309	102	AIX97831	complete ORF	18	gb|AIX97831.1| chemosensory protein [*Cnaphalocrocis medinalis*]	2e-70	100
CmedCSP13	363	120	AIX97832	complete ORF	16	gb|AIX97832.1| chemosensory protein [*Cnaphalocrocis medinalis*]	4e-68	100
CmedCSP14	327	108	AIX97833	complete ORF	18	gb|AIX97833.1| chemosensory protein [*Cnaphalocrocis medinalis*]	3e-59	100
CmedCSP15	381	126	AIX97834	complete ORF	18	gb|AIX97834.1| chemosensory protein [*Cnaphalocrocis medinalis*]	6e-75	100
CmedCSP16	435	144	AIX97835	complete ORF	18	gb|AIX97835.1| chemosensory protein [*Cnaphalocrocis medinalis*]	3e-76	100
CmedCSP17	474	157	AIX97836	complete ORF	18	gb|AIX97836.1| chemosensory protein [*Cnaphalocrocis medinalis*]	1e-75	100
CmedCSP18	363	120	AIX97837	complete ORF	15	gb|AIX97837.1| chemosensory protein [*Cnaphalocrocis medinalis*]	6e-83	100
CmedCSP19	354	117	AIX97838	complete ORF	16	gb|AIX97838.1| chemosensory protein [*Cnaphalocrocis medinalis*]	2e-80	100
CmedCSP21	387	128	AIX97840	complete ORF	18	gb|AIX97840.1| chemosensory protein [*Cnaphalocrocis medinalis*]	9e-81	100
CmedCSP33	363	120	ALT31615	complete ORF	16	gb|ALT31615.1| chemosensory protein 33 [*Cnaphalocrocis medinalis*]	1e-70	99
CmedCSP36	366	121	APY22695	complete ORF	18	gb|APY22695.1| chemosensory protein 36 [*Cnaphalocrocis medinalis*]	2e-83	99
CmedCSP37*	369	122	evm.TU.000005F.100	complete ORF	18	gb|APY22695.1| chemosensory protein 36 [*Cnaphalocrocis medinalis*]	3e-53	73
CmedCSP38*	462	153	evm.TU.000005F.84	complete ORF	17	gb|BAV56818.1| chemosensory protein 14 [*Ostrinia furnacalis*]	6e-71	67

Note: The newly identified CmedCSPs are marked with *.

Table S4. List of candidate odorant receptors in *C. medinalis*.

Gene name	Length (bp)	ORF (aa)	Accession number	Status	TMD	BLASTX best hit (Acc. no./Gene name/species)	E-value	Identity(%)
CmedOrco	1422	473	ALT31679	complete ORF	7	gb|ALT31679.1|odorant receptor co-receptor [*Cnaphalocrocis medinalis*]	0.0	100
CmedPR1	1128	375	ALT31681	complete ORF	5	gb|ALT31681.1|pheromone receptor 1 [*Cnaphalocrocis medinalis*]	0.0	98
CmedOR1	1389	462	ALT31655	complete ORF	6	gb|ALT31655.1|odorant receptor 1 [*Cnaphalocrocis medinalis*]	0.0	100
CmedOR2	1224	407	ALT31656	complete ORF	7	gb|ALT31656.1|odorant receptor 2 [*Cnaphalocrocis medinalis*]	0.0	100
CmedOR3	1296	431	ALT31657	complete ORF	6	gb|QEE82767.1|odorant receptor 49 [*Conogethes pinicolalis*]	0.0	79
CmedOR4	1800	599	ALT31658	complete ORF	7	gb|BAR43451.1|putative olfactory receptor 9 [*Ostrinia furnacalis*]	3e-158	72
CmedOR5	1206	401	ALT31659	complete ORF	7	gb|QIJ45811.1|olfactory receptor [*Glyphodes pyloalis*]	0.0	61
CmedOR6	795	264	ALT31660	complete ORF	4	gb|ALT31660.1|odorant receptor 6 [*Cnaphalocrocis medinalis*]	5e-88	93
CmedOR7	1296	431	ALT31661	complete ORF	6	gb|QEE82767.1|odorant receptor 49 [*Conogethes pinicolalis*]	0.0	75
CmedOR8	1170	389	ALT31662	complete ORF	7	gb|ALT31662.1|odorant receptor 8 [*Cnaphalocrocis medinalis*]	2e-125	95
CmedOR9	1260	419	ALT31663	complete ORF	5	gb|QIJ45783.1|olfactory receptor [*Glyphodes pyloalis*]	0.0	73
CmedOR10	1107	368	ALT31664	complete ORF	5	gb|ALT31664.1|odorant receptor 10 [*Cnaphalocrocis medinalis*]	1e-128	100
CmedOR14	1071	356	ALT31668	complete ORF	5	gb|QEE82755.1|odorant receptor 37 [*Conogethes pinicolalis*]	0.0	75
CmedOR16	1161	386	ALT31670	complete ORF	6	gb|QIJ45806.1|olfactory receptor [*Glyphodes pyloalis*]	0.0	73
CmedOR17	1161	386	ALT31671	complete ORF	6	gb| BAR43452.1|putative olfactory receptor 10 [*Ostrinia furnacalis*]	0.0	69
CmedOR18	1167	388	ALT31672	complete ORF	7	gb| ARO76436.1|odorant receptor 31 [*Conogethes punctiferalis*]	0.0	75
CmedOR19	1206	401	ALT31673	complete ORF	5	gb| ARO76432.1|odorant receptor 27 [*Conogethes punctiferalis*]	0.0	91
CmedOR20	1290	429	ALT31674	complete ORF	7	gb| ARO76440.1|odorant receptor 35 [*Conogethes punctiferalis*]	0.0	92
CmedOR22	1176	391	ALT31676	complete ORF	4	gb|QIJ45784.1|olfactory receptor [*Glyphodes pyloalis*]	0.0	68
CmedOR25	1299	432	ANZ03138	complete ORF	7	gb|ANZ03138.1|olfactory receptor 25 [*Cnaphalocrocis medinalis*]	0.0	99
CmedOR27	1308	435	ANZ03140	complete ORF	4	gb|ANZ03140.1|olfactory receptor 27 [*Cnaphalocrocis medinalis*]	0.0	98
CmedOR29	492	163	ANZ03142	complete ORF	3	gb|ANZ03142.1|olfactory receptor 29 [*Cnaphalocrocis medinalis*]	8e-71	98
CmedOR30	1140	379	ANZ03143	complete ORF	6	gb|ANZ03143.1|olfactory receptor 30 [*Cnaphalocrocis medinalis*]	0.0	99
CmedOR31	1227	408	ANZ03144	complete ORF	3	gb|ANZ03144.1|olfactory receptor 31 [*Cnaphalocrocis medinalis*]	0.0	98
CmedOR32	525	174	ANZ03145	3′ missing	4	gb|ANZ03145.1|olfactory receptor 32 [*Cnaphalocrocis medinalis*]	7e-122	100
CmedOR33	1221	406	ANZ03146	complete ORF	6	gb|ANZ03146.1|olfactory receptor 33 [*Cnaphalocrocis medinalis*]	0.0	100
CmedOR35	1248	415	ANZ03148	complete ORF	7	gb|ARO76445.1|odorant receptor 40 [*Conogethes punctiferalis*]	0.0	67
CmedOR37	414	137	ANZ03150	complete ORF	3	gb|ANZ03150.1|olfactory receptor 37 [*Cnaphalocrocis medinalis*]	4e-69	100
CmedOR38	1200	399	ANZ03151	complete ORF	6	gb|XP_028158571.1|odorant receptor Or2-like [*Ostrinia furnacalis*]	0.0	73
CmedOR39	1251	416	ANZ03152	complete ORF	4	gb|ANZ03152.1|olfactory receptor 39 [*Cnaphalocrocis medinalis*]	0.0	100

CmedOR40	1272	423	ANZ03153	complete ORF	5	gb|ANZ03153.1|olfactory receptor 40 [*Cnaphalocrocis medinalis*]	0.0	100
CmedOR41	963	320	ANZ03154	complete ORF	5	gb|ANZ03154.1|olfactory receptor 41 [*Cnaphalocrocis medinalis*]	1e-137	68
CmedOR43	873	290	ANZ03156	complete ORF	4	gb|ANZ03156.1|olfactory receptor 43 [*Cnaphalocrocis medinalis*]	0.0	85
CmedOR45	1269	422	ANZ03158	3′ missing	5	gb|ANZ03158.1|olfactory receptor 45 [*Cnaphalocrocis medinalis*]	0.0	99
CmedOR46	660	219	ANZ03159	complete ORF	3	gb|ANZ03159.1|olfactory receptor 46 [*Cnaphalocrocis medinalis*]	6e-93	99
CmedOR47	1200	399	ANZ03160	3′ missing	6	gb|QEE82743.1|odorant receptor 25 [*Conogethes pinicolalis*]	1e-78	37
CmedOR49	1296	431	ANZ03162	complete ORF	6	gb|QIJ45802.1|olfactory receptor [*Glyphodes pyloalis*]	4e-170	61
CmedOR51	1200	399	ANZ03164	complete ORF	6	gb|QIJ45795.1|olfactory receptor [*Glyphodes pyloalis*]	0.0	78
CmedOR54*	1221	406	novel.3382	complete ORF	7	gb|ANZ03139.1|olfactory receptor 26 [*Cnaphalocrocis medinalis*]	0.0	68
CmedOR55*	1110	369	evm.TU.000497F.7	complete ORF	7	gb|QEE82748.1|odorant receptor 30 [*Conogethes pinicolalis*]	0.0	72
CmedOR56*	1212	403	evm.TU.000145F.8	complete ORF	6	gb|BAR43464.1|putative olfactory receptor 22 [*Ostrinia furnacalis*]	0.0	74
CmedOR57*	1209	402	novel.3252	3′ missing	6	gb|ANZ03139.1|olfactory receptor 26 [*Cnaphalocrocis medinalis*]	0.0	69
CmedOR58*	1110	369	evm.TU.000497F.6	complete ORF	6	gb|QIJ45814.1|olfactory receptor [*Glyphodes pyloalis*]	2e-164	62
CmedOR59*	1194	397	evm.TU.000027F.21	complete ORF	6	gb|QEI46825.1 |odorant receptor 9 [*Galleria mellonella*]	2e-99	46
CmedOR60*	1170	389	evm.TU.000092F.9	complete ORF	5	gb| BAR43474.1|putative olfactory receptor 32 [*Ostrinia furnacalis*]	1e-87	44
CmedOR61*	1185	394	evm.TU.000027F.21.1	3′ missing	5	gb| ARO76443.1|odorant receptor 38 [*Conogethes punctiferalis*]	3e-168	65
CmedOR62*	1179	392	evm.TU.000180F.17	3′ missing	4	gb| ANZ03159.1|olfactory receptor 46 [*Cnaphalocrocis medinalis*]	5e-118	61
CmedOR63*	795	264	evm.TU.000671F.3	complete ORF	4	gb| ARO76437.1|odorant receptor 32 [*Conogethes punctiferalis*]	4e-78	46
CmedOR64*	837	278	evm.TU.000247F.7	complete ORF	3	gb| ARO76412.1|odorant receptor 6 [*Conogethes punctiferalis*]	1e-51	56
CmedOR65*	477	158	evm.TU.000409F.3	complete ORF	3	gb| QIJ45798.1|olfactory receptor [*Glyphodes pyloalis*]	5e-79	80
CmedOR66*	552	183	evm.TU.000761F.1	complete ORF	2	gb| ARO76421.1|odorant receptor 15 [*Conogethes punctiferalis*]	2e-87	72
CmedOR67*	654	217	novel.2055	complete ORF	2	gb| QIJ45826.1|olfactory receptor [*Glyphodes pyloalis*]	7e-64	50
CmedOR68*	879	292	evm.TU.001076F.2	complete ORF	1	gb|QIJ45817.1|olfactory receptor [*Glyphodes pyloalis*]	1e-88	62
CmedOR69*	246	81	evm.TU.000409F.4	3′ missing	1	gb|ARO76419.1|odorant receptor 13 [*Conogethes punctiferalis*]	3e-38	84
CmedOR70*	366	121	novel.4585	complete ORF	0	gb|AZB49441.1|olfactory receptor 27 [*Heortia vitessoides*]	1e-70	89

Note: The newly identified CmedORs are marked with *.

Table S5. List of candidate ionotropic receptors in *C. medinalis*.

Gene name	Length (bp)	ORF (aa)	Accession number	Status	TMD	BLASTX best hit (Acc. no./Gene name/species)	E-value	Identity(%)
CmedIR8a	2706	901	ALT31619	complete ORF	3	gb|ALT31619.1| ionotropic receptor 1 [*Cnaphalocrocis medinalis*]	0.0	99
CmedIR25a	2313	770	APY22696	complete ORF	2	gb|XP_050348568.1| ionotropic receptor 25a isoform X2 [*Nymphalis io*]	0.0	80
CmedIR93a	2625	874	ALT31632	complete ORF	4	gb|ALT31632.1| ionotropic receptor 93a [*Cnaphalocrocis medinalis*]	0.0	100
CmedIR21a.2	948	315	ALT31624	complete ORF	3	gb|QEE82780.1|ionotropic receptor 6 [*Conogethes pinicolalis*]	0.0	93
CmedIR40a	1617	538	APY22697	5′ missing	3	gb|APY22697.1|ionotropic receptor IR40a [*Cnaphalocrocis medinalis*]	0.0	100
CmedIR41a	1620	539	ALT31625	complete ORF	2	gb|QIJ45772.1| ionotropic receptor [*Glyphodes pyloalis*]	0.0	75
CmedIR60a	1974	657	APY22698	complete ORF	3	gb|APY22698.1| ionotropic receptor IR60a [*Cnaphalocrocis medinalis*]	0.0	99
CmedIR64a	1800	599	APY22699	complete ORF	3	gb|APY22699.1|ionotropic receptor IR64a [*Cnaphalocrocis medinalis*]	0.0	100
CmedIR68a	2103	700	ALT31626	complete ORF	4	gb|QEE82777.1| ionotropic receptor 3 [*Conogethes pinicolalis*]	0.0	87
CmedIR75d	1803	600	APY22700	complete ORF	3	gb|QEE82787.1|ionotropic receptor 75d [*Conogethes pinicolalis*]	0.0	80
CmedIR75p.1	1737	578	ALT31628	complete ORF	2	gb|ALT31628.1| ionotropic receptor 75p [*Cnaphalocrocis medinalis*]	0.0	78
CmedIR75p.2	1206	401	ALT31620	complete ORF	2	gb|QEE82789.1| ionotropic receptor 75p1 [*Conogethes pinicolalis*]	0.0	69
CmedIR75q.1	804	267	ALT31627	complete ORF	2	gb|ALT31627.1| ionotropic receptor 75 [*Cnaphalocrocis medinalis*]	2e-174	100
CmedIR87a	1956	651	ALT31631	complete ORF	3	gb|ARO76464.1|ionotropic receptor 1 [*Conogethes punctiferalis*]	0.0	90
CmedIR1*	1824	607	evm.TU.000705F.5	complete ORF	1	gb|QIJ45778.1| ionotropic receptor [*Glyphodes pyloalis*]	0.0	77
CmedIR1.2*	1992	663	evm.TU.000047F.47	complete ORF	3	gb|AOE48004.1| ionotropic receptor IR1.2 [*Athetis lepigone*]	0.0	57
CmedIR7d.1*	1929	642	evm.TU.000078F.13	complete ORF	3	gb|QHB15314.1| ionotropic receptor 7d.1 [*Peridroma saucia*]	0.0	47
CmedIR7d.2.1*	1308	435	evm.TU.000078F.14	complete ORF	3	gb|KOB72673.1| Ionotropic receptor [*Operophtera brumata*]	1e-22	49
CmedIR7d.2.2*	1734	577	evm.TU.000078F.15	complete ORF	4	gb|QEI46861.1|ionotropic receptor 7d [*Galleria mellonella*]	0.0	49

Note: The newly identified CmedIRs are marked with *.

Table S6. List of candidate sensory neuron membrane proteins in *C. medinalis*.

Gene name	Length (bp)	ORF (aa)	Accession number	Status	TMD	BLASTX best hit (Acc. no./Gene name/species)	E-value	Identity(%)
CmedSNMP1	1578	525	AFG73002	complete ORF	2	gb|AFG73002.1| sensory neuron membrane protein 1 [*C. medinalis*]	0.0	100
CmedSNMP2	1563	520	AFG73003	complete ORF	1	gb|AFG73003.1| sensory neuron membrane protein 2 [*C. medinalis*]	0.0	99
